# Clinical features of carriers of reciprocal chromosomal translocations involving chromosome 2: report of nine cases and review of the literature

**DOI:** 10.1590/S1677-5538.IBJU.2017.0233

**Published:** 2018

**Authors:** Xinyue Zhang, Hongguo Zhang, Cong Hu, Ruixue Wang, Qi Xi, Ruizhi Liu

**Affiliations:** 1 Center for Reproductive Medicine and Center for Prenatal Diagnosis, First Hospital, Jilin University, Changchun, China

**Keywords:** Infertility, Male, Chromosomes, Human, Pair 2, Genetic Counseling

## Abstract

**Objective:**

To explore the clinical features of carriers of chromosome 2 translocations, enabling informed genetic counseling of these patients.

**Materials and Methods:**

Eighty-two male carriers of a translocation who were infertile or receiving fertility counseling were recruited. Cytogenetic analyses were performed using G-banding. A search of PubMed was performed to determine whether the identified translocations on chromosome 2 are involved in male infertility. The relationships of translocation breakpoints with male infertility and recurrent pregnancy loss were analyzed.

**Results:**

Of the 82 translocation carriers, 9 (11%) were carriers of a chromosome 2 translocation. Four cases had oligozoospermia or infertility, while five had normal semen. In an analysis of the literature, 55 patients who were carriers of chromosome 2 translocations were also reviewed. Breakpoints at 2p13 and 2q31 were observed in six patients each, and were the most common. Breakpoints at 2p23, 2p13, 2p11.2, 2q31, and 2q37 were associated to both pre-gestational and gestational infertility, while other breakpoints were associated with gestational infertility.

**Conclusions:**

All breakpoints at chromosome 2 were correlated with gestational infertility. Carriers of chromosome 2 translocations should therefore receive counseling to continue with natural conception and use of different technologies available via assisted reproductive technology, such as preimplantation genetic diagnosis.

## INTRODUCTION

Infertility affects approximately 15%-20% of couples who attempt to have children. Reciprocal translocations are present in 0.9/1000 newborns, and the incidence in the infertile male population is 7-10 times higher than in fertile men (1, 2). Balanced translocation is the most common structural rearrangement in humans (3). Chromosomal translocations may cause the loss of genetic material at the breakpoints and could result in testicular failure (4). Individuals affected by such translocations are associated with reproductive problems such as infertility, recurrent pregnancy loss, and malformed offspring (5). These effects are related to the specific chromosomes and breakpoints involved in the translocation (6, 7). Some translocation breakpoints can disrupt the structure of an important gene, leading to male infertility (8).

The genetic counseling of male carriers of translocations remains challenging. Preimplantation genetic diagnosis (PGD) is a recommended part of such counseling for those with balanced translocation with normal or abnormal semen. In vitro fertilization accompanied by PGD increases the chance of their fathering a healthy child (9). In azoospermia patients, pregnancy success and fertility may be achieved via intracytoplasmic sperm injection, using spermatozoa obtained from testis by microdissection testicular sperm extraction (10, 11).

However, De Krom et al. (12) reported that clinical characteristics including spontaneous abortion do not differ between those couples who accept and those who decline PGD. A systematic review also showed a lack of sufficient evidence that PGD improves the live birth rate in couples with repeated miscarriage carrying a structural chromosome abnormality (13). In addition, the natural pregnancy success rates for couples in which the male carries a translocation ranges from 30% to 70% (14). This suggests that continuing attempts to conceive naturally are a viable option for successful pregnancy. Hence, the relationship between chromosome structure abnormality and clinical features warrants further studies.

There may be important genes associated with spermatogenesis on chromosome 2. For example, follicle-stimulating hormone receptor (FSHR) is located on chromosome 2p16.3, and is expressed in testicular tissue of idiopathic azoospermic patients with severe spermatogenic defects. Its differential expression may be associated with the degree of spermatogenesis (15). A study has also shown that genetic polymorphisms in the FSHR gene might increase the susceptibility to azoospermia in Iranian men (16). However, the FSHR polymorphisms at the studied sites were shown not to be associated with idiopathic male infertility or to influence FSH levels in both normal and infertile males in the Han-Chinese population (17). In addition, the SPAG16 gene (sperm-associated antigen 16), mapped on chromosome 2 at 2q34, has been reported to be associated with impaired sperm motility (18). The breakpoints of 2q25.1, q11.2, and q31 have also been shown to be related to impaired spermatogenesis (19).

The present study was established to explore the clinical features and translocation breakpoints in carriers of reciprocal chromosomal translocations involving chromosome 2. This study also highlights the importance of genetic counseling for infertile patients.

## MATERIALS AND METHODS

### Study subjects

Between July 2010 and December 2015, we recruited 82 male carriers of translocations experiencing infertility, or receiving counseling, from the outpatient's department at the Centre for Reproductive Medicine, the First Hospital of Jilin University, Changchun, China. All patients underwent a thorough physical examination and semen analysis, and were required to complete a detailed questionnaire pertaining to their smoking habits, marital status, medical history, and working conditions. The study protocol was approved by the Ethics Committee of the First Hospital of Jilin University, and written informed consent was obtained from all participants.

### Semen analysis

Semen analysis was performed according to the procedures recommended by the World Health Organization guideline. If no sperm was found, sperm was analyzed by sedimentation of semen samples through centrifugation. Patients with oligozoospermia were diagnosed as a sperm cell count <15×10^6^/mL in their last 3 semen samples (taken at intervals of 1-3 weeks). Azoospermia and oligozoospermia were defined as previously described (8). All analyzes were performed at the same laboratory, and all data were accessed from medical records.

### Cytogenetic analysis

Cytogenetic analysis was carried out on all patients. Peripheral blood (0.5mL) was collected in sterile tubes containing 30U/mL heparin. Lymphocytes were then cultured in appropriate culture medium (Yishengjun; Guangzhou Baidi Biotech, Guangzhou, China) for 72h, and subsequently treated with colcemid for 1h. G-banding of meta-phase chromosomes and karyotype analysis were performed using previously described methods (20). Twenty metaphases were counted and 6 karyotypes were analyzed for per patient. The karyotype nomenclature was described in accordance of ISCN 2009. The resolution level of the chromosome analysis was 400-550 band levels.

### Analysis of the identified translocation breakpoints

A search for the translocations identified in chromosome 2 from infertile males was performed using PubMed. The keywords were “chromosome/translocation/sperm” and “chromosome/translocation/abortion” for Pubmed search. The criteria were that the patients included reciprocal chromosomal translocations involving chromosome 2 in reported papers. The relationships of translocation breakpoints with male infertility and recurrent pregnancy loss were analyzed. Such searches were performed for a total of 46 carriers of chromosomal 2 translocations. This study included the cases of reciprocal chromosomal translocations involving chromosome 2 in reported papers and excluded the cases without breakpoints involving chromosome 2.

## RESULTS

A total of 82 translocation carriers were detected in this study. Of these, nine (11%) were carriers of a chromosome 2 translocation. Karyotype results from these nine patients are summarized in Table-1. Four cases had oligozoospermia or infertility (pre-gestational infertility), while five cases had normal semen. Of these latter five cases, it was evident that their partners were able to conceive, but had a tendency to miscarry (gestational infertility): one case had experienced recurrent spontaneous abortions, one case had experienced two stillbirths, and one case had experienced biochemical pregnancy on three occasions, while two cases produced a phenotypically normal child.

**Table 1:** Karyotypes of chromosome 2 translocation carriers and their clinical features.

Infertility causes	Clinical findings	Karyotype
Pre-gestational infertility	Oligozoospermia or infertility	46,XY,t(1;2)(q21;p23) 46,XY,t(1;2)(q21;q37) 46,XY,t(2;13)(q10;q10) 46,XY,t(2;15)(p11.2;q15)
Gestational infertility	Normal sperm density; a history of miscarriage, stillbirth, or normal fertility	46,XY,t(2;6)(q21;p21) 46,XY,t(2;11)(q33;q23) 46,XY,t(2;11)(q35;q13) 46,XY,t(2;14)(q31;q24) 46,XY,t(2;16)(p23;q13)

An analysis of the literature was also performed, from which karyotype results, clinical manifestations, and the breakpoints on chromosome 2 were collected, as shown in Table-2. Breakpoints at 2p13 and 2q31 were observed in six patients each, and were the most common. Breakpoints at q10 and q11.2 were related to pre-gestational infertility, while breakpoints at 2p23, 2p13, 2p11.2, 2q31, and 2q37 were connected to both pre-gestational and gestational infertility. Other breakpoints were associated with gestational infertility. It is noteworthy that two carriers of a translocation at 2q33 produced normal children, as did one carrier of a translocation at 2q35 (Table-3).

**Table 2:** Breakpoints in chromosome 2 translocation carriers and clinical features.

Case	Karyotype	Breakpoints	Clinical findings	Reference
1	t(1;2)	1p22;2q31	2 miscarriages	Dong et al., 2014 (31)
2	t(1;2)	1q21;2p23	Oligozoospermia	The present study
3	t(1;2)	1q21;2q37	Oligozoospermia	The present study
4	t(1;2)	1q21;2q37	Oligozoospermia	Li et al., 2012 (23)
5	t(1;2)	1q32;2q36	Abortion	Templado et al., 1990 (32)
6	t(1;2)	1q32.1;2q11.2	Oligozoospermia	Vozdova et al., 2013 (9)
7	t(1;2)	1q42;2q37	2 fetal malformations	Zhang et al., 2006 (33)
8	t(1;2)	1q42;2q33	Miscarriage	Stasiewicz-Jarocka et al., 2000 (34)
9	t(2;3)	2p13;3q27	Recurrent pregnancy loss	Ocak et al., 2013 (35)
10	t(2;3)	2q21; 3p21	Recurrent spontaneous abortion	Tunç et al., 2016 (26)
11	t(2;3)	2q24;3p26)	Normal semen	Martin, 1994 (36)
12	t(2;4)	2p21;4p14	Recurrent abortion	Portnoï et al., 1988 (37)
13	t(2;4)	2q31;4q31	Stillbirth	Li et al., 2012 (23)
14	t(2;4)	2q31;4q31	2 stillbirths	Dong et al., 2014 (31)
15	t(2;5)	2p21;5p15	Recurrent spontaneous abortion	Gada Saxena et al., 2012 (25)
16	t(2;5)	2p13;5p15	Recurrent fetal wastage	Fryns et al., 1998 (38)
17	t(2;5)	2p11;5q15	Abortion	Templado et al., 1988 (39)
18	t(2;5)	2p11;5q31	Recurrent abortion	Portnoï et al., 1988 (37)
19	t(2;5)	2q12;5q35.3	Spontaneous miscarriage	Kochhar et al., 2013 (40)
20	t(2;6)	2p13;6p21.3	Recurrent abortion	Al-Hussain et al., 2000 (41)
21	t(2;6)	2p12;6q24	Two earlier miscarriages	Lim et al., 2003 (42)
22	t(2;6)	2q21;6p21	3 first-trimester abortions	The present study
23	t(2;6)	2q34;6p24	Recurrent spontaneous abortion	Gada Saxena et al., 2012 (25)
24	t(2;7)	2p23;7p22	Recurrent fetal wastage	Fryns et al., 1998 (38)
25	t(2;7)	2p13;7q34	Normal semen, IVF/PGD ET, twins 46,XX	Vozdova et al., 2013 (9)
26	t(2;7)	2p13;7q32	Primary infertility, abnormal semen	Vozdova et al., 2013 (9)
27	t(2;7)	2q31;7q34	Abnormal semen	Vozdova et al., 2013 (9)
28	t(2;7)	2p11.2;7q22.1	2 miscarriages	Wiland et al., 2008 (3)
29	t(2;7)	2q37.3;7q34)	3 miscarriages	Ahn et al., 2003 (43)
30	t(2;8)	2p13;8q13	Multiple spontaneous abortions	Castle et al., 1988 (44)
31	t(2;8)	2p22;8p23.1	Spontaneous abortions	Kyu Lim et al., 2004 (45)
32	t(2;8)	2q35;8q11.2	Spontaneous abortions	Kyu Lim et al., 2004 (45)
33	t(2;8)	2q37;8q22	3 abortions	Campana et al., 1986 (46)
34	t(2;9)	2q21;9p22	Abortion	Martin et al., 1990 (47)
35	t(2;9)	2q37.3;9q12	Normal semen, increased risk of miscarriage	Dul et al., 2012 (48)
36	t(2;10)	2q33;10q24	Produced a child, 46,XX	Faed et al., 1982 (49)
37	t(2;11)	2p14;11q21	Repeated abortion	Ananthapur et al., 2014 (5)
38	t(2;11)	2q24;11q32	Recurrent abortion	Al-Hussain et al., 2000 (41)
39	t(2;11)	2q33;11q23	Produced a child, 46,XX	The present study
40	t(2;11)	2q35;11q13	Produced a child, 46,XX,t(2;11)	The present study
41	t(2;12)	2q31;12q24	3 first-trimester abortions	Niroumanesh et al., 2011 (50)
42	t(2;13)	2q35;13q32	Repeated miscarriage	Goddijn et al., 2004 (51)
43	t(2;13)	2q10;13q10	Oligozoospermia	The present study
44	t(2; 14)	2p23.1;14q31	Abortion	Rousseaux et al., 1995 (52)
45	t(2;14)	2q12;14q32.33	Recurrent spontaneous abortion	Manvelyan et al., 2007 (27)
46	t(2;14)	2q31;14q24	2 stillbirths	The present study
47	t(2;14)	2q37.1;14q31.2	Fetal malformations	de la Fuente et al., 1988 (53)
48	t(2;15)	2p11.2;15q15	Infertility	The present study
49	t(2;15)	2q21;15p12	Recurrent fetal wastage	Fryns et al., 1998 (38)
50	t(2;16)	2p23;16q13	Recurrent spontaneous abortion	The present study
51	t(2;17)	2q35;17p13	Abortion	Jenderny, 1992 (54)
52	t(2;17)	2q37.2; 17q25	Recurrent spontaneous abortion	Bruyere et al., 2003 (55)
53	t(2;18)	2p21;18q11.2	5 miscarriages	Estop et al., 1995 (56)
54	t(2;20)	2p16;20p12	Recurrent abortion	Sachs et al., 1985 (57)
55	t(2;20)	2p24.1;20q13.1	Recurrent abortion	Trappe et al., 2002 (58)

**Table 3:** Incidence of breakpoints on chromosome 2.

Breakpoints	Number of patients with pre-gestational infertility	Number of patients with gestational infertility	Reproductive outcomes
p24.1		1	
p23.1		1	
p23	1	2	
p22		1	
p21		3	
p16		1	
p14		1	
p13	1	4	1 case of IVF/PGD ET, twins 46,XX
p12		1	
p11.2	1	1	
p11		2	
q10	1		
q11.2	1		
q12		2	
q21		4	
q24		2	
q31	1	5	
q33		1	2 cases of natural pregnancy, birth 46,XX
q34		1	
q35		3	1 case of natural pregnancy, birth 46,XX,t(2;11)
q36		1	
q37	2	2	
q37.1		1	
q37.2		1	
q37.3		2	
p24.1		1	

## DISCUSSION

Karyotype analysis is able to detect chromosomal translocations or deletions, which sometimes have very detrimental effects on gene structure, and remains a powerful and cheap method to use (21). This technology thus provides valuable information for the genetic counseling of infertile males (22). Previous studies have reported that infertile men have an 8-10-fold higher prevalence of chromosomal abnormalities than fertile men (23). Chromosomal translocation alters the complex and vital process of spermatogenesis, and leads to recurrent pregnancy loss (24). In particular, chromosome 2 translocation has often been associated with male infertility and recurrent miscarriage (9, 25, 26). In the present study, nine of our cases were identified as carriers of chromosome 2 translocations, and 55 cases of chromosome 2 translocation from the literature were also reviewed.

Generally, male infertility can be broadly divided into two types of reproductive failure: pre-gestational and gestational infertility (23). In this study, the breakpoints that we identified on chromosome 2 were found to be associated with pre-gestational or gestational infertility. Four cases were associated with pre-gestational infertility and five cases were related to gestational infertility. Kim et al. (19) reported that the breakpoints at 2p25.1, 2q11.2, and 2q31 could interfere with spermatogenesis, and that the breakpoint at 2p13 was related to recurrent abortion. In addition, Manvelyan et al. (27) reported that the breakpoint at 2q12 in male carriers was associated with repeated abortion. To study the relationship of these breakpoints on chromosome 2 with male infertility, we analyzed recent published literature and revealed clinical features in carriers of chromosome 2 translocations. The karyotype results and clinical findings at chromosome 2 are summarized in Table-2. Clinical features associated with the breakpoints at 2p13, 2q11.2, and 2q12 were consistent with the above two reports (19, 27).

Table-3 also shows that 2p23, 2p13, 2p11.2, 2q31, and 2q37 were connected to both pre-gestational and gestational infertility. These cases indicated that these breakpoints are not responsible for pre-gestational infertility, so another breakpoint of translocation must be the cause in these individuals. Similarly, the APOB gene is located on chromosome 2p24.1, and the APOB gene signal peptide deletion polymorphism was reported not to be associated with infertility in Indian men (28). Furthermore, FSHR, mapped on chromosome 2 at 2p16, was shown not to be correlated with sperm count in infertile males (29).

Besides the breakpoints at q10 and q11.2, other breakpoints were identified as being associated with gestational infertility. For those affected by these breakpoints, natural conception is possible and they have the potential to bear normal children. For example, Ikuma et al. (30) reported that the live birth rate with natural conception for translocation carriers was 37%-63% in the first trial and 65%-83% cumulatively. However, natural conception is still a greater risk, since the number of chromosomal unbalanced gametes is large, leading to repetitive pregnancy loss, which may have repercussions on the fertility of the translocation carrier. For these carriers, informed choice should be performed. In addition, the breakpoints at 2p13 and 2q31 were found to be the most common, and were associated with gestational infertility.

## CONCLUSIONS

In the present study, 55 carriers of chromo-some 2 translocations were reviewed. The breakpoints at 2p13 and 2q31 were the most common, and were associated with gestational infertility. All breakpoints at chromosome 2 were correlated with gestational infertility. Carriers of chromosome 2 translocations should therefore be counselled to attempt natural conception and to use the different technologies available via assisted reproductive technology, such as PGD.

## Abbreviations

PGD: =Preimplantation genetic diagnosis
FSH: =Follicle-stimulating hormone
FSHR: =Follicle-stimulating hormone receptor
SPAG16 gene: =Sperm-associated antigen 16 gene
APOB gene: =Apolipoprotein B gene
